# Tuning Photophysical Properties of Donor/Acceptor Hybrid Thin- Film via Addition of SiO_2_/TiO_2_ Nanocomposites

**DOI:** 10.3390/polym13040611

**Published:** 2021-02-18

**Authors:** Bandar Ali Al-Asbahi, Mohammad Hafizuddin Hj. Jumali, M. S. AlSalhi, Saif M. H. Qaid, Amanullah Fatehmulla, Wafa Musa Mujamammi, Hamid M. Ghaithan

**Affiliations:** 1 Department of Physics & Astronomy, College of Sciences, King Saud University, Riyadh 11451, Saudi Arabia; malsalhi@ksu.edu.sa (M.S.A.); sqaid@ksu.edu.sa (S.M.H.Q.); aman@ksu.edu.sa (A.F.); walmujammi@ksu.edu.sa (W.M.M.); 436107632@student.ksu.edu.sa (H.M.G.); 2 Department of Physics, Faculty of Science, Sana’a University, Sana′a 12544, Yemen; 3 School of Applied Physics, Faculty of Science and Technology, Universiti Kebangsaan Malaysia, Selangor, Bangi 43600, Malaysia; hafizhj@ukm.edu.my; 4 Research Chair in Laser Diagnosis of Cancers, College of Sciences, King Saud University, Riyadh 11451, Saudi Arabia; 5 Department of Physics, Faculty of Science, Ibb University, Ibb 70270, Yemen

**Keywords:** nanocomposite, donor/acceptor, optical properties, energy transfer, thin films

## Abstract

The influence of SiO_2_/TiO_2_ nanocomposites (STNCs) content on non-radiative energy transfer (Förster-type) from poly (9,9′-dioctylfluorene-2,7-diyl) (PFO) to poly [2-methoxy-5-(2-ethylhexyloxy)-1,4-phenylenevinylene] (MEH-PPV) using steady-state and time-resolved photoluminescence spectroscopies was investigated at room temperature. The improved energy transfer from PFO to MEH-PPV upon an increment of the STNCs was achieved by examining absorbance, emission (PL) and photoluminescence excitation (PLE) spectra. The shorter values of the quantum yield (φ_DA_) and lifetime (τ_DA_) of the PFO in the hybrid thin films compared with the pure PFO, indicating efficient energy transfer from PFO to MEH-PPV with the increment of STNCs in the hybrid. The energy transfer parameters can be tuned by increment of the STNCs in the hybrid of PFO/MEH-PPV. The Stern–Volmer value (k_SV_), quenching rate value (k_q_), Förster radius (R_0_), distance between the molecules of PFO and MEH-PPV (R_DA_), energy transfer lifetime (τ_ET_), energy transfer rate (k_ET_), total decay rate of the donor (TDR), critical concentration (A_o_), and conjugation length (A_π_) were calculated. The gradually increasing donor lifetime and decreasing acceptor lifetime, upon increasing the STNCs content, prove the increase in conjugation length and meanwhile enhance in the energy transfer.

## 1. Introduction

The incorporation of inorganic nanostructures into the organic materials imparted interesting optoelectronic properties to nanocomposite materials and opened a new way for designing unique optical-based materials for optoelectronic applications. One of the most significant approaches to improve the efficiency of the optoelectronic devices, such as organic light emitting diodes (OLEDs), is through fabricated emissive layers based on donor/acceptor combinations [1,2,3]. The improvement of the optoelectronic properties and thus OLEDs emission efficiency can be achieved by controlling the donor/acceptor content and shifting the emission wavelength away from the donor absorption band by a non-radiative energy transfer from the donor excited state to that of the acceptor [4,5]. Despite the significant attention of such approach, intermolecular interaction can occur in the donor/acceptor combination and thus quench the device luminescence [6]. The incorporation of inorganic nanostructures into the donor/acceptor hybrid was successfully employed to overcome such problem and thus improved the device performance [2,7,8]. Recently, it was found that the incorporation of SiO_2_/TiO_2_ nanocomposites (STNCs) into the conjugated polymers plays a crucial role in improvement of OLED devices performance, where the STNCs possess the features of both SiO_2_ and TiO_2_ NPs [9,10].

Numerous approaches, such as solution blending method [2], sol –gel [11], in situ polymerization [12], and star-like polymer as nanoreactors [13], have been established to prepare well-defined organic/inorganic nanocomposites. In the recent studies, the effect of fixed ratio of individual TiO_2_ or SiO_2_ NPs and fixed STNCs on the energy transfer of the various blend ratios of poly [2methoxy-5-(2-ethyl-hexyloxy)-1,4-phenylenevinylene] (MEH-PPV) and poly (9,9′-di-n-octylfluorenyl-2.7-diyl) (PFO) was investigated in details [7]. The ultraviolet and visible emission intensities of STNCs were greater than that of pristine TiO_2_ or SiO_2_ NPs, subsequently, the addition of the STNCs into the hybrids of the donor/acceptor enhanced the energy transfer properties [14].

Despite several reports representing the effect of incorporation TiO_2_ or SiO_2_ NPs on the physical properties of Donor/acceptor hybrid, there have been no reports on the effect of STNCs content on the tuning photophysical properties of PFO/MEH-PPV. In the current work, MEH-PPV (as acceptor) and PFO (as donor) were blended at fixed ratio and the tuning optical properties and the energy transfer parameters by various weight ratios of the STNCs will be demonstrated here in terms of quantum yield (φ_DA_), lifetime (τ_DA_), Stern–Volmer value (k_SV_), quenching rate value (k_q_), Förster radius (R_0_), distance between the molecules of donor and acceptor (R_DA_), energy transfer lifetime (τ_ET_), energy transfer rate (k_ET_), the total decay rate of the donor (TDR), the critical acceptor concentration (A_o_), the conjugation length (A_π_), and lifetime decay for the emission regions of donor and acceptor. 

## 2. Materials and Methods

PFO and MEH-PPV with weight-average molecular weight 58,200 and 40,000 g/mol, respectively were purchased from Sigma Aldrich (Saint Louis, Missouri, USA) and were used as received without further purification. The SiO_2_ NPs in amorphous phase was synthesized by sol-gel method, as reported in our previous study [15], and mixed with anatase TiO_2_ NPs (which was purchased from Sigma Aldrich) to form SiO_2_/20 wt% TiO_2_ NCs (STNCs) as prepared in our previous report [14]. The fixed ratio of PFO/10 wt% MEH-PPV hybrid, in a toluene solvent produced by Fluka, was prepared with various weight ratios of STNCs: 5, 10, 15, and 20 wt% by solution blending method. A spin coating technique with 2000 rpm for 20 s was used to deposit 70 μL from each sample onto a glass substrate (1.2 cm × 2 cm). All samples were annealed at 120 °C in a vacuum oven to remove the solvent. 

To collect the both absorption and emission spectra as well as lifetime decays, Perkin Elmer Lambda 900 ultraviolet–visible Spectrometer and an Edinburgh Instrument FLSP920 spectrophotometer were used respectively. The thin films morphology was characterized by field emission scanning electron microscope (FE-SEM) (Zeiss Supra 55VP, Oberkochen, Germany).

## 3. Results

### 3.1. Optical Properties

**Figure 1:**
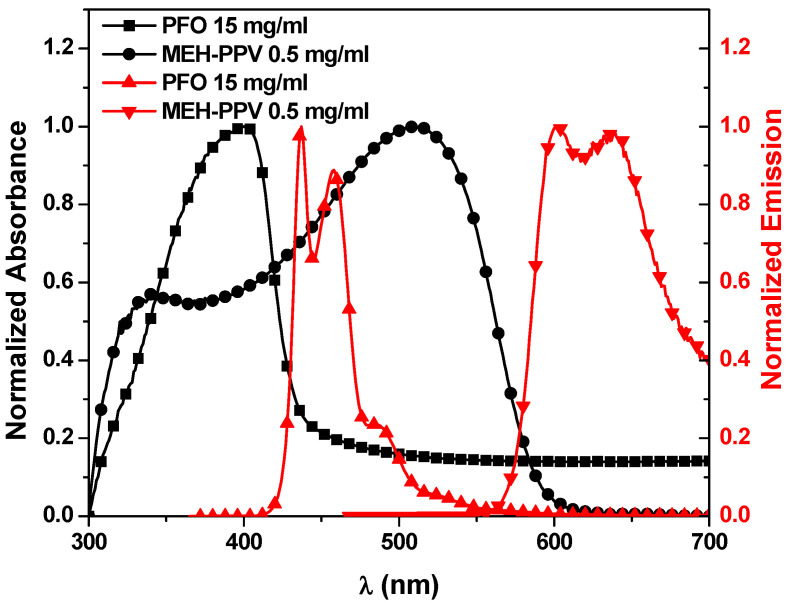
Normalized absorbance (black color) and emission spectra (red color) of PFO and MEH-PPV thin films.

The normalized absorbance and emission spectra of pristine PFO and pristine MEH-PPV are shown in Figure 1. One absorbance peak at 397 nm corresponding to π→π* transition appeared for PFO besides two emission peaks at 437 nm and 458 nm referring to 0→0 and 0→1 transition respectively, and a shoulder at 488 nm referring to 0→2 transition. On the other side, two absorbance peaks at 339 nm and 508 nm appeared for MEH-PPV corresponding to 0→1 and 0→0 transition respectively, and two emission peaks at 600 nm and 637 nm referring to 0→0 and 0→1 transition respectively. 

**Figure 2:**
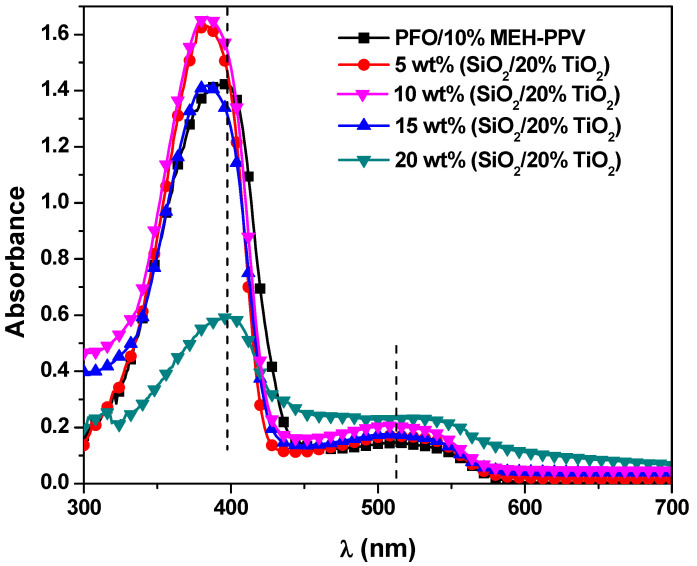
Absorbance spectra of PFO/MEH-PPV hybrid thin films with various ratios of STNCs.

The influence of the STNCs content on the absorbance of the donor/acceptor hybrid thin films is displayed in Figure 2. Two absorbance peaks at 394 nm and 512 nm can be observed referring to that of PFO and MEH-PPV respectively. It can be noticed that comparing with pristine conjugated polymers shown in Figure 1, the first peak (394 nm) was blue shifted ~3 nm whereas the second (512 nm) red shifted ~ 4 nm without appearing any new peaks, indicating no chemical interaction occurs [16]. Upon increment of STNCs into the PFO/10 wt% MEH-PPV hybrid, the absorbance of the first peak enhanced and blue shifted up to 14 nm, whereas the second peak improved and dramatically red shifted up to ~33 nm. Opposite trend was observed for the first peak with high content of the STNCs (>15 wt%) and new peak at ~ 310 nm referring to 0→1 transition of pure MEH-PPVwith blue shift by ~ 29 nm. This means that the incorporation content of the STNCs in the hybrid is limited to improve both of the absorbance peaks related to both PFO and MEH-PPV. Moreover, the increasing absorbance with slightly blue shifting of the first peak that related to PFO is a strong indication that the STNCs have an effect on the conjugation length of the PFO [17] which will be corroborated later in Section 3.2.4.

**Figure 3:**
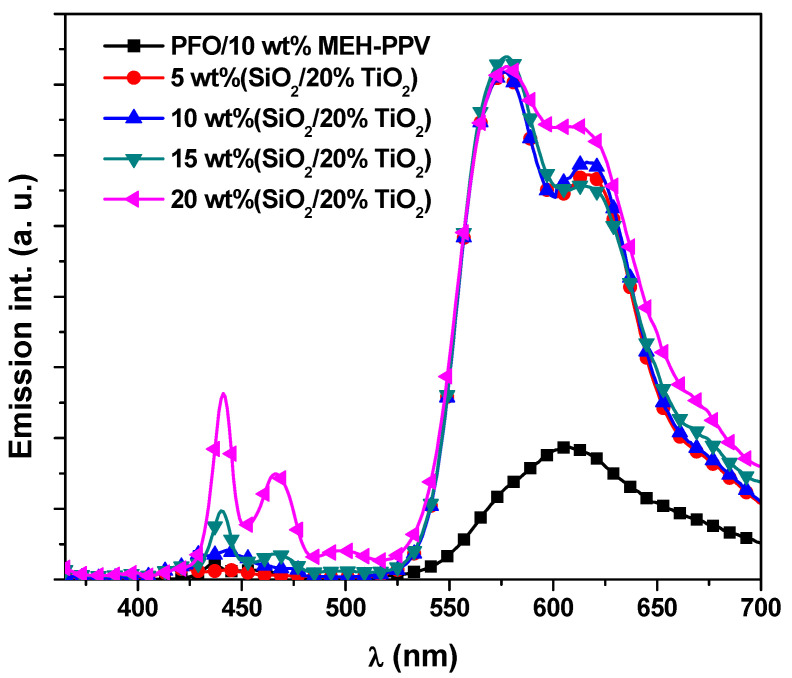
Emission spectra of PFO/MEH-PPV hybrid thin films with various ratios of STNCs.

**Figure 4:**
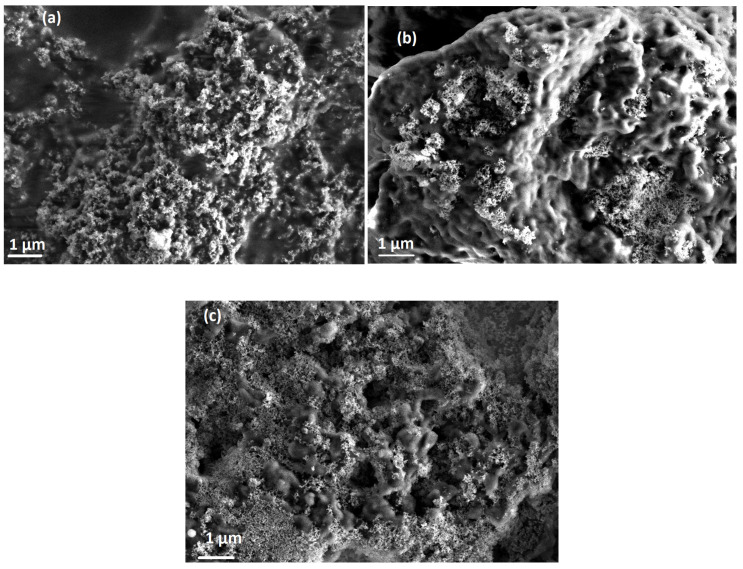
FE-SEM images of PFO/10 wt% MEH-PPV hybrid thin film with: (**a**) 10 wt% STNCs, (**b**) 15 wt% STNCs, and (**c**) 20 wt% STNCs.

Figure 3 shows emission spectra of PFO/MEH-PPV hybrid thin films with various ratios of STNCs photoexcited by 355 nm. Upon the inclusion of STNCs into the hybrids, a significant improvement in emission intensity from both PFO and MEH-PPV was detected. The MEH-PPV exhibited much faster intensity enhancement compared to PFO. It is reasonable to deduce that the significant enhanced emission intensity (Figure 3) and weak absorption of MEH-PPV in the hybrid thin films (Figure 2) is mainly due to improved energy transfer from PFO to MEH-PPV with increment the STNCs. In conjunction with the gradual enhancement of the three emission peaks that related to the PFO upon increment the STNCs, the emission peak of the MEH-PPV referring to the 0-0 transition was enhanced with fixed intensity even the content of the STNCs increased, whereas that peak for 0-1 transition was dramatically improved. As found in recent report [14], the STNCs with ratio of STNCs exhibited emission in the region 350–525 nm. Consequently, the enhancement in the emission intensity at region 400-525 nm referring to PFO in the PFO/MEH-PPV hybrids with the STNCs, resulted from the radiative transitions and oxygen vacancies that comes from the STNCs. Oxygen vacancies can act as an electron trap, resulting in enhancing the emission properties of the hybrid thin films by improving the UV light absorption. As a result, this enhancement leads to improve the emission intensity that is related to MEH-PPV through energy transfer mechanism from the PFO to MEH-PPV. Moreover, many studies proposed that the emission improvement in inorganic/conjugated polymers nanocomposites is mainly attributable to the formation of more extended chains and then increase the polymer conjugation length in addition to charge trapping effect [18,19,20,21]. On the other hand, the enhancing emission may result from the agglomeration of STNCs on the surface of the PFO/MEH-PPV hybrid, as seen in FE-SEM images (Figure 4).

**Figure 5:**
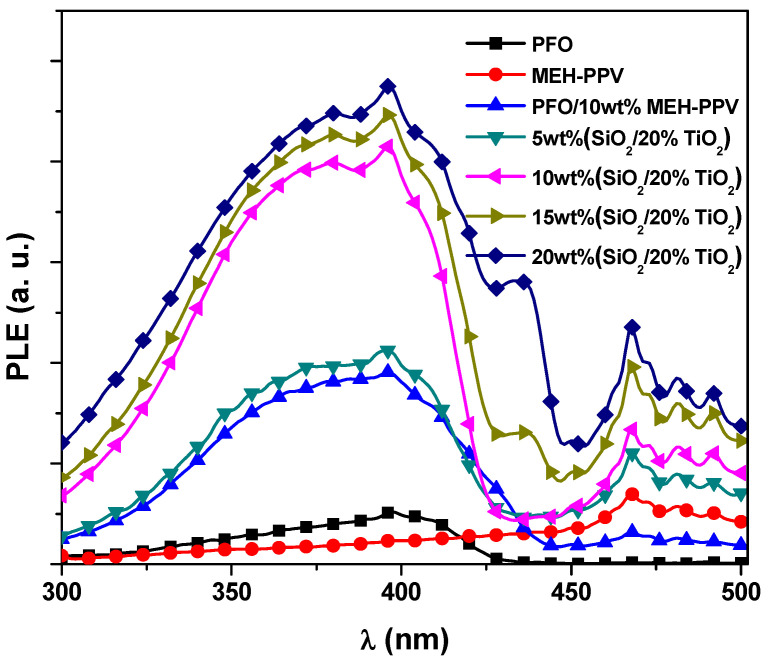
PLE spectra of the PFO, MEH-PPV, and PFO/MEH-PPV with various ratios of STNCs collected at the monitoring emission wavelength of 550 nm.

The improved energy transfer from PFO to MEH-PPV upon increment the STNCs can be achieved by examining photoluminescence excitation (PLE) spectra. Figure 5 displays the PLE results acquired from the pristine PFO, pristine MEH-PPV, and PFO/10 wt% MEH-PPV hybrid with different STNCs contents collected at the monitoring emission wavelength of 550 nm. The very low PLE intensity of the PFO under the excitation wavelength (355 nm) and the very weak emission intensity at 550 nm in the emission spectrum of the PFO (see Figure 1), indicating that the emission at 550 nm mainly causes from MEH-PPV in the PFO/MEH-PPV hybrid. The PLE intensity peak at 355 nm increases obviously upon increment the STNCs content which is consistent with the emission intensity from MEH-PPV shown in the emission spectra of the PFO/MEH-PPV with increasing the STNCs content. These results indicate that both photo-excitation in PFO and the content of STNCs contribute to the emission from MEH-PPV in the hybrid thin films. As the PFO/MEH-PPV hybrid with various content of STNCs are photo-excited by the 355 nm, the intrachain excimers can be formed in PFO during their lifetimes in addition to the charge trapping effect. Consequently, simultaneous enhanced emission from MEH-PPV and PFO is observed and then energy transfer from PFO to MEH-PPV improved.

### 3.2. Energy Transfer Parameters

As found in recent report [7] at ratio of 10 wt% MEH-PPV in the hybrid thin film of PFO/MEH-PPV, the donor emission was quenched and the emission intensity of the acceptor concurrently reduced instead of increasing. This observation suggested that the energy transfer from the PFO to the significant number of MEH-PPV molecules was converted into heat, where these molecules acted as dark quenchers without any fluorescence. In the current work, a remarkable improvement in emission intensity from both PFO and MEH-PPV was verified with the inclusion of STNCs into the hybrid of PFO/10 wt% MEH-PPV (Figure 3). The MEH-PPV displayed much higher intensity improvement compared to PFO, conforming the improved energy transfer in present the STNCs. 

The energy transfer parameters for the PFO/MEH-PPV hybrid with the STNCs was obtained at excitation wavelength of 355 nm which direct MEH-PPV excitation was insignificant and energy transfer from the PFO to the MEH-PPV occurred. In the sections below, numerous parameters were estimated to describe the influence of the STNCs on the energy transfer mechanism.

#### 3.2.1. Quantum Yield and Lifetime of the Donor in the Hybrids

The tunable quantum yield (φ*_DA_*) and lifetime (τ*_DA_*) values of the PFO in the hybrid thin films was achieved by the addition of STNCs using the following formula [22]:

(1) $$\frac{I_{D}}{I_{DA}}=\;\frac{\tau_{D}}{\tau_{DA}}=\;\frac{\varphi_{D}}{\varphi_{DA}}$$

where *I_D_* and *I_DA_* sign to the emission intensities of donor in the absence and presence of both quenchers (STNCs and acceptor), respectively.

The φ*_DA_* was varied from 0.0137 to 0.2650 whereas the τ*_DA_* from 6.57 ps to 127 ps with the increment of STNCs as tabulated in Table 1. However, these values of φ*_DA_* and τ*_DA_* still shorter than that of pure PFO thin film (φ*_D_* = 0.72 and τ*_D_* = 346 ps) [1], indicating the efficient energy transfer from PFO to MEH-PPV upon increment the STNCs in the hybrid.

#### 3.2.2. Stern–Volmer (k_SV_) and Quenching Rate (kq) Constants

The Stern–Volmer value (k_SV_) of the donor/acceptor hybrid was moderated with addition of the STNCs using the following formula [23]:

(2) $$\frac{I_{D}}{I_{DA}}=1+k_{SV}\left[A\right]$$

where [*A*] is the acceptor concentration.

The values of k_SV_ dramatically decreased from 1.24 to 0.041 (μM)^−1^ as summarized in Table 1. These values implied that 50% of the emission was quenched for the acceptor concentration (A_1/2_) increases systematically from approximately 0.81 to 24.3 μM with increasing the STNCs (Table 1).

The quenching rate value ($k_{q}=\frac{k_{SV}}{\tau_{D}}$) was tuned by the incorporation of STNCs into the PFO/MEH-PPV hybrid, from 3.57 × 10^15^ to 0.119 × 10^15^ M^−1^.S^−1^ (Table 1). Since the minimum value for efficient quenching is 1 × 10^10^ M^−1^.S^−1^ [22], the high values of *k_q_* in the current work indicate the STNCs well mixing with the PFO/MEH-PPV hybrid. 

#### 3.2.3. Förster Radius, Energy Transfer Rate, and Energy Transfer Lifetime

**Figure 6:**
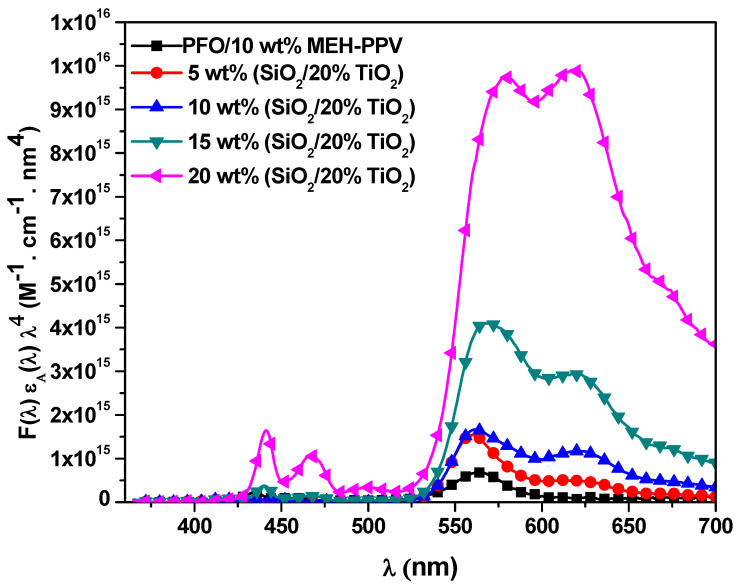
*F*(λ) ε(λ) λ^4^ versus wavelength for PFO/MEH-PPV with various ratios of STNCs.

The integral values under the curves in Figure 6 were employed to calculate the critical distance of the energy transfer, *R*_0_ (Förster radius) using the following equation:

(3) $$R_{o}^{6}\;=\frac{5.89\times 10^{-5}\phi_{D}}{n^{4}}\;J\left(\lambda \right)$$

where *n* is the solvent refractive index [22,24]. The values of both J(*λ*) = $\int F\left(\lambda \right){\;\varepsilon }_{A}\left(\lambda \right){\;\lambda }^{4}d\lambda$ and *R*_0_ are presented in Table 2. With the addition of STNCs, the value of the *J*(λ) significantly increased from 0.82 × 10^15^ to 21.8 × 10^15^ M^−1^. cm^−1^.nm^4^ and thus the *R*_0_ tuned from 43.7 to 75.6 Å, which still in the range of 10–100 Å that confirm the efficient long-range dipole-dipole energy transfer (Förster type) upon increment the STNCs in the hybrid thin film [25,26].

The distance between the molecules of PFO and MEH-PPV (R_DA_) was calculated based on the Förster radius and emission intensities of the donor in absence and presence the acceptor using the following equation [27]:

(4) $$1-\frac{I_{DA}}{{\;I}_{D}}=\frac{R_{o}^{6}}{R_{DA}^{6}+R_{o}^{6}}$$

As presented in Table 2, when the STNCs content increased from 0 to 20 wt%, the *R_DA_* increased from 22.6 to 69.0 Å.

Additional evidence for the efficient incorporation of the STNCs on the energy transfer in the hybrid PFO/MEH-PPV can be found from the tunable values of energy transfer lifetime (τ*_ET_*), total decay rate of the donor (TDR = *k_ET_* + τ*_D_*^−1^), and energy transfer rate (*k_ET_*), which are calculated using the following expressions [22] and listed in Table 2.

(5) $$\tau_{ET}=\frac{1}{\left[A\right]k_{q}}\;\&\;k_{ET}=\frac{1}{\tau_{D}}{\left(\frac{R_{o}}{R_{DA}}\right)}^{6}$$

The τ*_ET_* increased while the *k_ET_* and TDR reduced with increment the STNCs into the donor/acceptor hybrid. This finding can be attributed to the increase in the distance between PFO (donor) and MEH-PPV molecules (acceptor) [28].

#### 3.2.4. Critical Concentration of Acceptor (A_o_) and Conjugated Length (A_π_)

The concentration of the MEH-PPV should be lower than the critical concentration ($A_{0}=\frac{447}{R_{o}^{3}}$), which is the concentration of the acceptor with 76% energy transfer [22], to suppress the intermolecular transfer in the PFO. The A_o_ values of MEH-PPV can be significantly tuned by incorporation the STNCs. As presented in Table 1, it was varied from 5.4 to 1.0 mM with increasing the STNCs content.

The distance between the dipoles arising from the ground state (S_0_) to the excited singlet state (S_1_) transition is defined as the conjugation length (A_π_) in the excited singlet state. It can be derived from the nonradiative rate constant (k_nr_) and radiative rate constant (k_r_) according to the following formula [29]:

(6) $$A_{\pi }=\ln\left(\frac{k_{r}}{k_{nr}}\right)$$

Upon increment of the STNCs, the value of k_r_ exhibits approximately no change (2.08 ns^−1^) while significant modification in the value of k_nr_ was observed, as listed in Table 1, indicating dominant non-radiative energy transfer and negligence radiative energy transfer. Therefore, A_π_ slightly increased with the increment of the STNCs. The increase in both values of R_DA_ and A_π_ with increment the STNCs showed that the STNCs increased the distance between the molecules of PFO and MEH-PPV.

**Figure 7:**
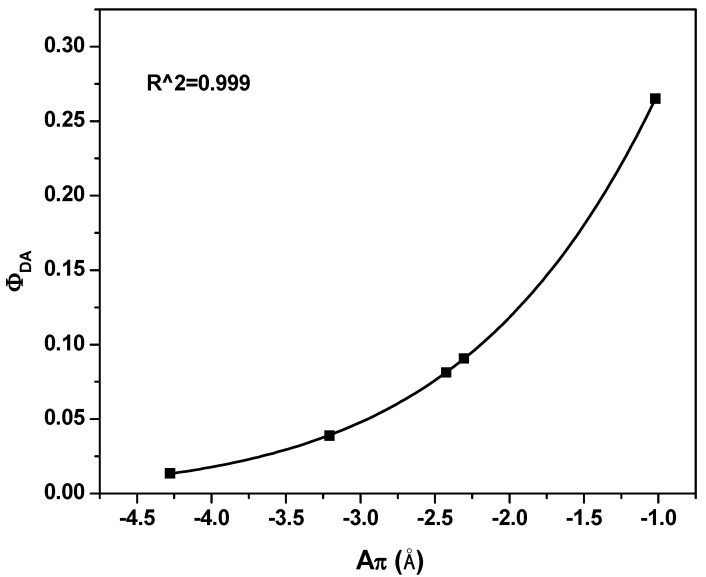
Fluorescence quantum yield versus the conjugated length for PFO/MEH-PPV with various ratios of STNCs.

The exponential increasing in φ_DA_ with A_π_ upon increment of the STNCs, as shown in Figure 7, demonstrate unique nanocomposites with high fluorescence as confirmed previously in Figure 3.

### 3.3. Lifetime Decay

The fluorescence lifetime (in Table 1) is differed from the lifetime decay that estimated in this section. The fluorescence lifetime includes the radiative and nonradiative emission, while the lifetime decay contains the radiative emission [24]. Both fluorescence lifetime and lifetime decay demonstrated more information about the influence the STNCs content on the interaction between PFO and MEH-PPV in the excited state.

**Figure 8:**
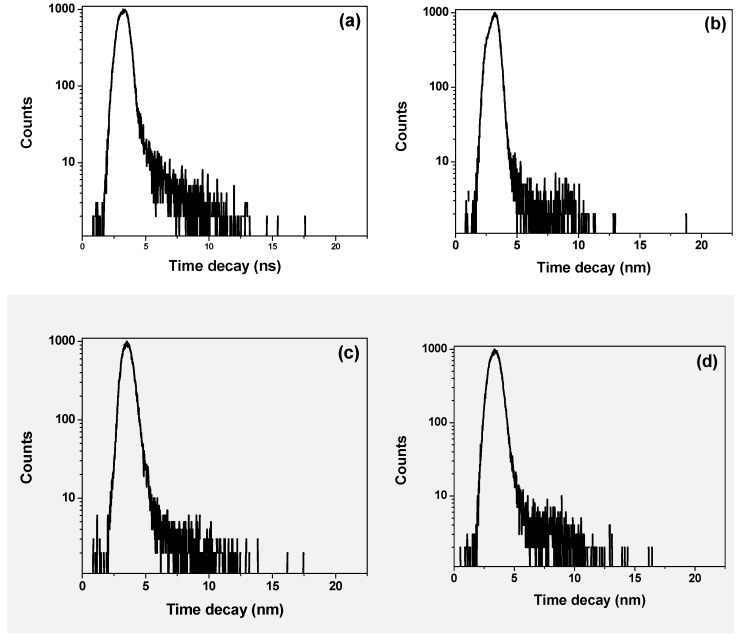

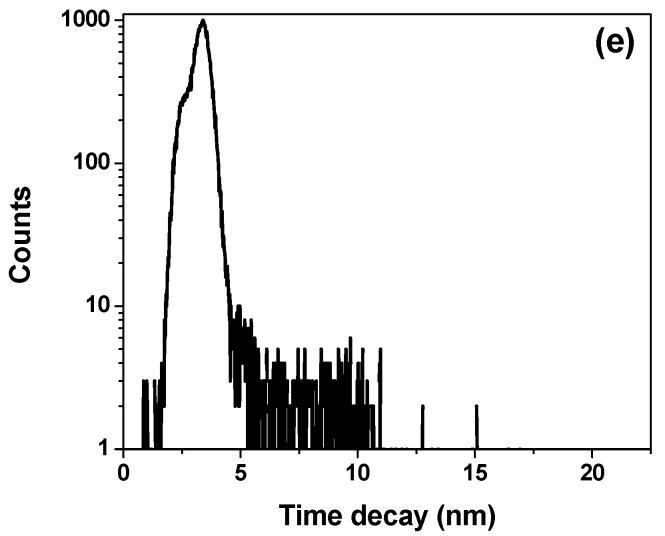
Lifetime decays of PFO/10 wt% MEH-PPV with various ratios of STNCs (**a**) 0, (**b**) 5 wt%, (**c**) 10 wt%, (**d**) 15 wt% & (**e**) 20 wt% at emission wavelength of 440 nm. λ_ex_= 376.4 nm.

**Figure 9:**
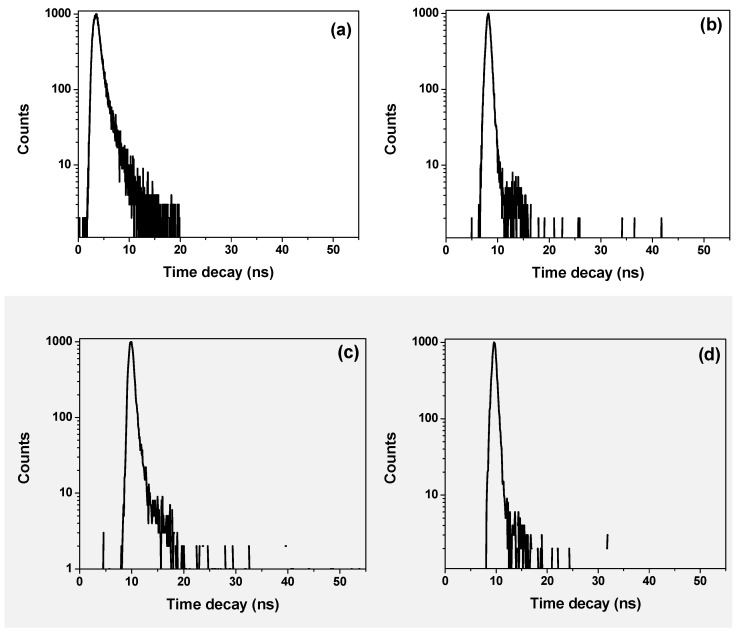

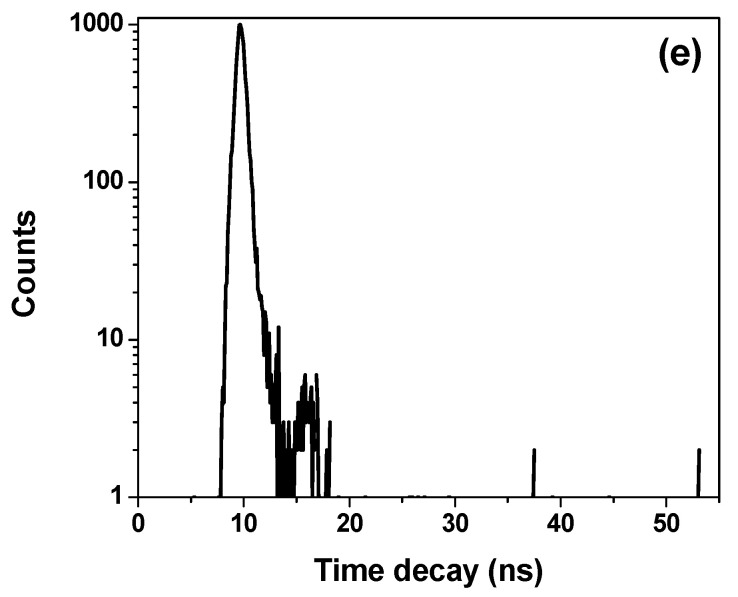
Lifetime decays of PFO/10 wt% MEH-PPV with various ratios of STNCs (**a**) 0, (**b**) 5 wt%, (**c**) 10 wt%, (**d**) 15 wt% & (**e**) 20 wt% at emission wavelength of 580 nm. λ_ex_ = 376.4 nm.

The lifetime decays obtained at 440 nm and 580 nm for the hybrid thin films with various STNCs content, refer to the emission regions of the donor and acceptor, respectively as shown in Figure 8a–e & Figure 9a–e. A comprehensive investigation including the data collected at 440 and 580 nm was also carried out, confirming the general trends of the data in Table 3. The main fitting parameters χ^2^, B_i_ and τ_i_ are presented in Figure 8 & Figure 9 and listed in Table 3 to estimate the effect of STNCs content on the lifetime decay of the hybrid thin film. It can be clearly observed that the donor lifetime (τ_D_) increased twice at 5 wt% STNCs content and then continue gradually increased with increment the STNCs. On the other hand, the acceptor lifetime (τ_A_) decreased around three times at 5 wt% STNCs content and then continue slight decrease with increment of the STNCs. Increasing donor lifetime proves the increase in conjugation length whereas decreasing the acceptor lifetime proves the enhance in the energy transfer, which are compatible with the previous findings. 

## 4. Conclusions

The modification in the photophysical properties of the donor/acceptor hybrid thin films was achieved by the incorporation of various STNCs content. The significant improved emission intensity of both PFO and MEH-PPV and weak absorption of MEH-PPV in the hybrid thin films refer to enhance energy transfer from PFO to MEH-PPV with an increment of STNCs. The shorter values of φ_DA_ and τ_DA_ compared with that of pure PFO thin film indicate the efficient energy transfer from PFO to MEH-PPV with addition of the STNCs. The Förster radius (R_0_) was tuned from 43.7 to 75.6 Å where RDA increased from 22.6 to 69.0 Å when the STNCs content increased from 0 to 20 wt%. The critical concentration varied from 5.4 to 1.0 mM while the conjugation length slightly increased from −4.278 to −1.020 Å with the increment of the STNCs from 0 to 20 wt%. Therefore, such donor/acceptor/STNCs composite is believed to be unique potential for designing new materials for optoelectronic devices.

## Figures and Tables

**Table 1:** Optical properties of PFO/10 wt% MEH-PPV hybrid thin film with various content of STNCs.

STNCs (wt%)	φ_DA_	τ_DA_ (ps)	k_nr_ (ns)^−1^	k_sv_ (µM)^−1^	k_q_ × 10^15^ (M.S)^−1^	A_π_ (Å)	A_0_ (mM)	A_1/2_ (µM)
0	0.0137	6.57	150.1	1.24	3.57	−4.278	5.4	0.81
5	0.0389	18.7	51.4	0.420	1.21	−3.207	3.7	2.38
10	0.0814	39.1	23.5	0.188	0.543	−2.424	2.9	5.32
15	0.0907	43.6	20.9	0.166	0.480	−2.305	1.8	6.02
20	0.2650	127	5.8	0.041	0.119	−1.020	1.0	24.3

**Table 2:** Energy Transfer Parameters of PFO/10 wt% MEH-PPV hybrid thin film with various content of STNCs.

STNCs (wt%)	J(λ) × 10^15^ (M^−1^.cm^−1^.nm^4^)	R_0_ (Å)	R_DA_ (Å)	k_ET_ (ns)^−1^	τ_ET_ (ps)	TDR (ns)^−1^
0	0.82	43.7	22.6	149	6.70	152
5	1.68	49.3	30.6	50.6	19.7	53.5
10	2.82	53.7	38.1	22.6	44.1	25.6
15	7.18	62.8	45.5	20.1	49.9	22.9
20	21.8	75.6	69.0	4.96	201	7.85

**Table 3:** Main fitting parameters and lifetime decays of PFO/10 wt% MEH-PPV hybrid thin film with various content of STNCs.

At λ_em._ 440 nm				At λ_em._ 580 nm					
STNCs (wt%)	Relative Amplitude	Donor Lifetime		Relative Amplitude		Acceptor Lifetime (ps)			
	B	τ_D_ (ps)	χ^2^	B_1_	B_2_	τ_1_	τ_2_	τ_A_ (ps)	χ^2^
0	0.316	60	1.784	0.049	0.004	461	1766	772	1.138
5	0.160	110	1.198	0.160	-	275	-	275	1.069
10	0.432	116	1.095	0.432	-	234	-	234	1.069
15	0.574	114	0.912	0.298	-	264	-	264	0.848
20	0.660	178	0.982	0.555	-	253	-	253	0.908

## Data Availability

The data presented in this study are available on request from the corresponding author.
